# Combined application of biochar and halophyte intercropping enhances cucumber yield and quality by ameliorating soil properties in a continuous cropping system

**DOI:** 10.3389/fpls.2025.1711099

**Published:** 2025-11-20

**Authors:** Songsong Shen, Yusheng Xu, Zhongpeng Liu, Yating Luo, Ruifang Wang, Guanlin Li, Yujing Liu

**Affiliations:** 1 School of tea and Coffee, Pu’er University, Puer, China; 2 Nanjing Agriculture University, College of Life Sciences, Nanjing, China; 3 School of Environment and Safety Engineering, Jiangsu University, Zhenjiang, China; 4 College of Tropical Crops, Hainan University, Haikou, China

**Keywords:** biochar, phytoremediation, soil salinity, nutrient imbalance, bacterial community, cucumber, continuous cropping

## Abstract

Biochar amendment and halophyte intercropping are viable strategies for alleviating soil degradation in greenhouse systems, specifically the secondary salinization and autotoxicity induced by continuous cropping. Nevertheless, the potential synergistic effects of combining these practices remain poorly understood. This study investigated their synergistic effects on soil properties, microbial communities, and cucumber performance. A pot experiment was conducted with the following treatments: soil without amendment (CK), biochar (B), *Paspalum vaginatum* intercropping (S), and biochar combined with *Paspalum vaginatum* intercropping. The results showed that BS treatment led to the highest increases in soil organic carbon content, pH, total nitrogen content, available phosphorus content, and available potassium content compared to CK (p<0.05). Concurrently, BS significantly reduced available nitrogen, electrical conductivity, Na^+^, SO_4_^2-^, and Cl^-^ levels, while total phosphorus remained unaffected. Cucumber yield increased significantly by 11.50% and 27.12% under B and BS treatments, respectively, whereas S showed no significant effect. BS also achieved the highest fruit quality enhancement, followed by B and S. Notably, B and S treatments displayed the highest and lowest K^+^, Ca^2+^and Mg^2+^ accumulation, respectively, whereas the BS treatment led to K^+^ and Ca^2+^ concentrations that were significantly lower than those in the B treatment. Soil bacterial diversity was significantly enhanced under BS. The PLS-PM identified the alleviation of soil salinity and acidity, along with improved nutrient availability, as the primary drivers for enhanced crop performance, with soil bacterial diversity playing a secondary yet significant role. These findings suggest that biochar combined with intercropping (BS) effectively mitigates continuous cropping obstacles in greenhouse systems by synergistically improving soil health and microbial ecology.

## 1 Introduction

Greenhouse cultivation offers a stable environment that meets the global demand for vegetables, making it one of the most widely adopted agricultural systems worldwide (Li et al., 2019; Lopez-Marin et al., 2019). Its high profitability often leads to continuous cropping and excessive fertilization (Liu and Zhang, 2021; Lv et al., 2019). However, continuous monoculture and excessive fertilizer input often lead to a decline in soil quality (Wang et al., 2021b; Yang et al., 2016), secondary soil salinization (Hu et al., 2020), soil acidification (Song et al., 2016; Zhang et al., 2022b), autotoxicity (Xiao et al., 2020), and the accumulation of soil-borne pathogens (Meng et al., 2018), which limit the yield and quality of greenhouse crops (Sun et al., 2019). Hence, implementing effective soil management strategies is crucial to maintaining the yield and quality of greenhouse crops, given the system’s global importance and the numerous soil degradation processes it induces.

Owing to its strong adsorption capacity and alkaline nature, biochar is considered a promising amendment for soil health management (Shi et al., 2023; Spokas et al., 2012). It has been shown to mitigate several continuous cropping obstacles simultaneously, such as by adsorbing allelopathic phenolic compounds (Lin et al., 2023), enhancing nutrient availability (Han et al., 2023; Yan et al., 2022), and optimizing the microbial habitat (Wang et al., 2020). However, a critical but often overlooked risk is that biochar application may also introduce or exacerbate soil salinity, primarily due to the direct input of salt ions present in the biochar itself, especially when derived from high-salt feedstocks (Amini et al., 2016; Wang et al., 2023b). Additionally, the aging process can diminish biochar’s adsorption capacity, potentially causing the re-release of previously bound salt ions (Akhtar et al., 2015; Kong et al., 2014). This inherent limitation suggests that biochar alone might be insufficient or even risky for managing the saline conditions often associated with greenhouse continuous cropping.

Conversely, halophytes represent a low-cost phytoremediation strategy widely employed in agriculture to improve saline soil conditions (Jurado et al., 2024; Liang and Shi, 2021). For example, intercropping with salt-tolerant species, such as lawn grass (*Paspalum vaginatum*) (Hu et al., 2020), *Portulaca oleracea* (Simpson et al., 2018), or legumes (Zheng et al., 2023), has been demonstrated to alleviate salt stress, improve crop quality and yield, and enhance soil nutrient availability. This approach effectively reduces the adverse effects of salinity on crops (Aksoy et al., 2001). Although effective for salinity control, the capacity of halophyte intercropping alone to rapidly improve broader soil issues like severe acidification or nutrient immobilization may be limited.

Thus, we hypothesize that integrating biochar amendment with halophyte intercropping could create a synergistic solution for the multifaceted challenges of continuous cropping. Biochar’s ability to rapidly adjust pH, improve nutrient retention, and adsorb phenolics could establish a more favorable base soil condition. This improved environment might, in turn, enhance the establishment and salt-uptake efficiency of the intercropped halophyte. Meanwhile, the halophyte can continuously remove salts from the soil, mitigating the potential salinization risk from biochar and preventing salt rebound. While both biochar and intercropping individually enhance soil health (Liu et al., 2024; Wang et al., 2023a), their combined effects, particularly the potential synergy in reshaping the soil microbial community to foster a more resilient and beneficial microbiome under the complex stress of continuous cropping, remain poorly understood (Jin et al., 2024). This knowledge gap is critical given the crucial role of soil microorganisms in maintaining soil health and suppressing soil-borne diseases (Gu et al., 2023; Liu et al., 2023a).

Therefore, this study aimed to investigate the potential of combined biochar amendment and intercropping with the halophyte *Paspalum vaginatum* as an integrated strategy to concurrently address multiple soil constraints (acidity, salinity, nutrient imbalance, and autotoxicity) in a continuous cucumber system. The specific objectives were to: (1) assess the effects of biochar and/or *Paspalum vaginatum* intercropping on cucumber growth and fruit quality; (2) evaluate the changes in key soil physicochemical properties induced by these treatments; and (3) investigate their collective influence on soil bacterial communities to identify the key factors determining cucumber performance. We hypothesized that the combined application would synergistically ameliorate soil properties, reshape the microbial community structure towards a more beneficial state, and consequently lead to greater improvements in cucumber yield and quality compared to either practice alone.

## 2 Materials and methods

### 2.1 Experimental setup

A pot experiment was conducted in a greenhouse at the teaching and research base of Nanjing Agricultural University, Nanjing, China (118°51' E, 32°01' N). The experimental soil (sandy loam) was collected from the top layer (0–20 cm) of a greenhouse in Jurong City, Jiangsu Province, China (119°13' E, 31°47' N), which had been under continuous cucumber monoculture for 15 years. The soil properties were as follows: pH, 5.43; electrical conductivity (EC), 1500 µS cm^-^¹; soil organic carbon (SOC), 5.91 g kg^-^¹; total nitrogen (TN), 1.41 g kg^-^¹; total phosphorus (TP), 1.28 g kg^-^¹. Biochar, supplied by Zhenjiang Zedi Biotechnology Co., Ltd. (Zhenjiang, China), was produced by anaerobic pyrolysis of rice straw at 600 °C. Its physicochemical characteristics were: pH, 8.85; SOC, 620 g kg^-1^; TN 10.5 g kg^-1^; available phosphorus (AP), 1.13 g kg^-1^; available potassium (AK), 23.1 g kg^-1^; Brunauer-Emmett-Teller (BET) surface area, 675 m^2^ g^-1^;and elemental composition of carbon (C), hydrogen (H), nitrogen (N), and oxygen (O) at 62.0%, 2.5%, 2.2%, and 20.6%, respectively. Cucumber seeds (cv. Xinjin No. 4) were purchased from Luming Seed Co., Ltd. (Taian, China). The *Paspalum vaginatum* was selected for intercropping based on research reporting its salt tolerance and ability to effectively alleviate secondary salinization in greenhouse soils (Hu et al., 2020).

A completely randomized design with four treatments was implemented: soil without amendment (CK), intercropping with *Paspalum vaginatum* (S), soil amended with biochar at 2% (w/w) (B), and 2% (w/w) biochar amendment combined with *P. vaginatum* intercropping. The biochar was thoroughly mixed with the soil and then placed into plastic pots (total volume of 6.5 L). Each treatment consisted of three independent biological replicates. To ensure robust sampling, each biological replicate comprised three pots, which were treated as technical replicates. Cucumber seeds were germinated in seedling trays. At the two-true-leaf stage, uniform and vigorous seedlings were transplanted into the pots (one seedling per pot). Concurrently, stem cuttings of *Paspalum vaginatum* were planted at a density of ten cuttings per pot. *Paspalum vaginatum* was trimmed to 4 cm height at 25 and 40 days after transplanting, and all trimmings were removed from the pots. Throughout the experiment, only cucumbers and *Paspalum vaginatum* were retained; weeds were manually removed. No fertilizers, herbicides, or pesticides were applied. Pests were managed using insect-proof nets and yellow sticky traps. All pots were irrigated equally every three days.

### 2.2 Soil sampling and analysis

After harvest, rhizosphere soil was collected using the root-shaking method (Inderjit and Mallik, 1997). The composite soil sample from each replicate was divided into three parts: one was stored at -80 °C for microbial DNA sequencing; one was stored at 4 °C for the measurement of available·nitrogen (sum of NH_4_^+^-N and NO_3_^-^-N) and the remainder air-dried for subsequent analysis of soil pH, EC, SOC, TN, TP, AP, AK, water-soluble ions, and phenolic compounds. For these analyses, measurements from the three technical pots within a biological replicate were averaged to yield a single value representing that replicate.

Soil pH and EC were measured in a 1:5 (w/v) soil-water suspension using a portable pH meter (FieldScout pH400, Spectrum Technologies Inc., USA) and a conductivity meter (DDSJ-308F, LeiCi, China), respectively. NH_4_^+^-N and NO_3_^-^-N were extracted with 2 M KCl at a soil-to-solution ratio of 1:5 (w/v) and analyzed with a flow injection auto-analyzer (AutoAnalyzer 3, Seal Analytical, Norderstedt, Germany). SOC was determined by the potassium dichromate oxidation method using a Multi N/C 2100 analyzer (Analytik Jena, Germany), after removing inorganic carbon by fumigation with concentrated HCl. TN and TP were determined using a flow injection autoanalyzer (AutoAnalyzer 3, Seal Analytical, Germany) following digestion with H_2_SO_4_-HClO_4_. AP was extracted with 0.5 M NaHCO_3_ (1:5, w/v) and analyzed using the flow injection autoanalyzer. AK was extracted with 1 M ammonium acetate (1:10, w/v) and quantified using a flame photometer (BWB Technologies Ltd., UK). Water-soluble ions were extracted with deionized water at a 1:5 (w/v) ratio. Cation (Na^+^, K^+^, Ca²^+^, Mg²^+^) concentrations were determined by inductively coupled plasma optical emission spectrometry (ICP-OES; iCAP 6300, Thermo Fisher Scientific, USA), and anion (Cl^-^, SO_4_²^-^) concentrations were measured by ion chromatography (ICS-5000, Thermo Fisher Scientific, USA). Total phenolics, complex phenolics, and water-soluble phenolics were determined according to the Folin-Ciocalteu method (Zhou et al., 2021).

### 2.3 Plant growth and fruit quality analysis

Plant height was measured 30 days after transplanting. At harvest, three random plants per treatment were selected. The total yield per plant was determined by harvesting all fruits from each selected plant. The aboveground biomass and roots of the cucumber plants were separately oven-dried at 65 °C until a constant weight was achieved. The vitamin C content was determined using the 2,6-dichloroindophenol titration method. Nitrate content was measured using the salicylic acid method (Zhang et al., 2020). Soluble sugar content was determined by extraction with boiling water followed by analysis using the anthrone colorimetric method (Pasin et al., 2020; Zhao et al., 2017).

### 2.4 DNA extraction and illumina HiSeq sequencing

Genomic DNA was extracted from soil samples using the E.Z.N.A. Soil DNA Kit (Omega Bio-tek, Inc., USA) according to the manufacturer’s instructions. The quality and concentration of the extracted DNA were confirmed using a Nanodrop 2000 (ThermoFisher Scientific, Inc., USA). Bacterial 16S rRNA gene V3-V4 regions were amplified using the universal primers 38F and 806R (Caporaso et al., 2011). An 8-bp barcode sequence was added to the 5’ end of both the forward and reverse primers to distinguish different samples. The final barcoded primers were used for amplification on an ABI 9700 PCR instrument (Applied Biosystems, Inc., USA). Polymerase chain reaction (PCR) was performed as previously reported (Yang et al., 2019b). Two rounds of PCR amplification were conducted, and the size of the amplified target bands was detected using 1% agarose gel electrophoresis. PCR products were purified using the Agencourt AMPure XP (Beckman Coulter, Inc., USA) nucleic acid purification kit.

### 2.5 Microbial community analysis

The sequencing data were demultiplexed based on the barcode sequences. The software Pear (v0.9.6) was used to filter and assemble the sequencing data, with a minimum overlap of 10 bp during assembly (Zhang et al., 2014). After assembly, sequences shorter than 230 bp were removed using the Vsearch (v2.7.1) software (Rognes et al., 2016), and chimeric sequences were identified and removed using the uchime method against the Gold Database (Edgar et al., 2011). The uparse algorithm in Vsearch (v2.7.1) was used to cluster high-quality sequences into operational taxonomic units (OTUs) with a sequence similarity threshold of 97% (Edgar, 2013). The representative sequences of OTUs were aligned against the Silva138 database using the BLAST algorithm, with an e-value threshold set at 1e-5, to obtain the taxonomic information for each OTU (Johnson et al., 2008; Pruesse et al., 2007).

Based on the OTU and abundance results, alpha diversity indices were calculated using the QIIME (v1.8.0) software, and plots were generated using R (v3.6.0) software (Caporaso et al., 2010). Bacterial community diversity was analyzed based on the Bray-Curtis dissimilarity index, and principal coordinates analysis (PCoA) plots were generated (Caporaso et al., 2010). For community composition visualization, the top 10 most abundant bacterial phyla were plotted based on their relative abundance; all remaining phyla were grouped into an ‘Other’ category. Biomarker features in each group were screened by Metastats and LEfSe software. Functional profiling of the soil microbial metagenome was predicted from the 16S rRNA gene sequencing data using PICRUSt based on Kyoto Encyclopedia of Genes and Genomes (KEGG) pathways.

### 2.6 Statistical analyses

Given the nested design, data from the three technical pots per biological replicate were averaged, resulting in a sample size of n = 3 per treatment for all statistical tests. All data were statistically analyzed using SPSS 22.0 (SPSS, Inc., Chicago, IL, United States). Significant differences (p < 0.05) among treatments, based on one-way ANOVA followed by Duncan’s test, are indicated by different lowercase letters. All column charts were created using Origin 2024b (OriginLab Corporation, Northampton, MA, USA). Partial least squares path model (PLS-PM) was used to determine the direct and indirect effects of soil factors pH, available N, soil nutrients index (total P, available P, available K), phenolic index (total phenols, complex phenolic, and water-soluble phenolic), properties (SO_4_^2-^, Na^+^) and soil bacteria on cucumber growth and quality. The reliability of the model was assessed using the Goodness of Fit (GoF) metric. A GoF value ≥ 0.36 indicates better model alignment (Wetzels et al., 2009).

## 3 Result

### 3.1 Effects of biochar and intercropping on cucumber growth

**Figure 1:**
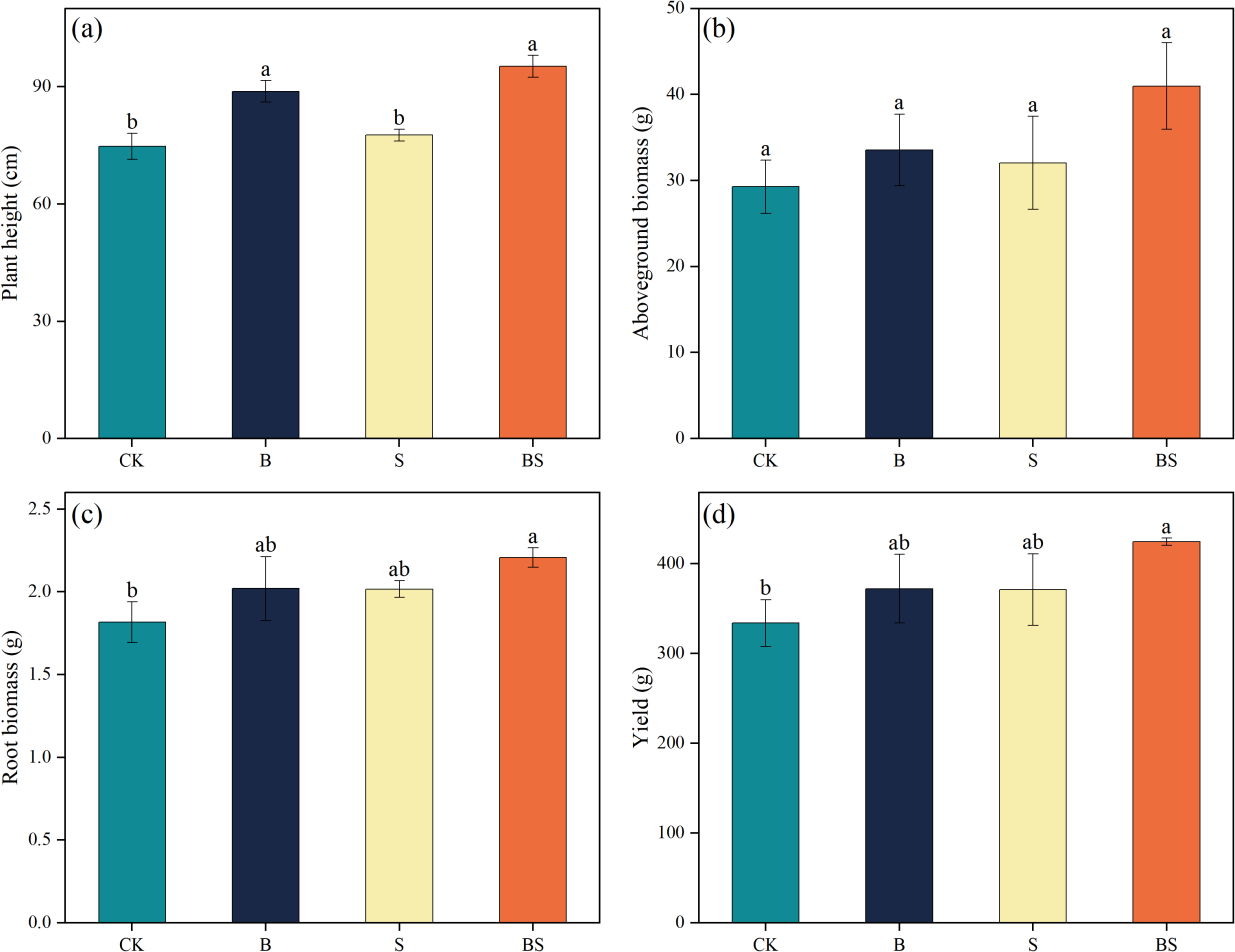
Effects of biochar and intercropping on plant height **(a)**, above-ground biomass **(b)**, below-ground biomass **(c)**, and yield **(d)** of continuously cropped cucumbers. Each value represents three biological replicates (± SD) (n = 3). Different lowercase letters indicate significant differences among treatments at the *p* < 0.05 level.

The application of biochar (B), intercropping with *Paspalum vaginatum* (S), and their combination promoted cucumber plant growth compared to CK (**Figure 1**). Plant height was significantly increased by treatments B and BS compared to CK, with the BS treatment achieving the greatest height among all treatments (**Figure 1a**). In contrast, above-ground biomass did not differ significantly among treatments (**Figure 1b**). Total root biomass increased by 11.19% (B), 10.92% (S), and 21.47% relative to CK (**Figure 1c**). Of these, only the increase in the BS treatment was statistically significant. Cucumber yield was also significantly increased by all treatments relative to CK, by 11.50% (B), 8.18% (S), and 27.12% (*p* < 0.05; **Figure 1d**).

### 3.2 Effects of biochar and intercropping on cucumber quality

**Figure 2:**
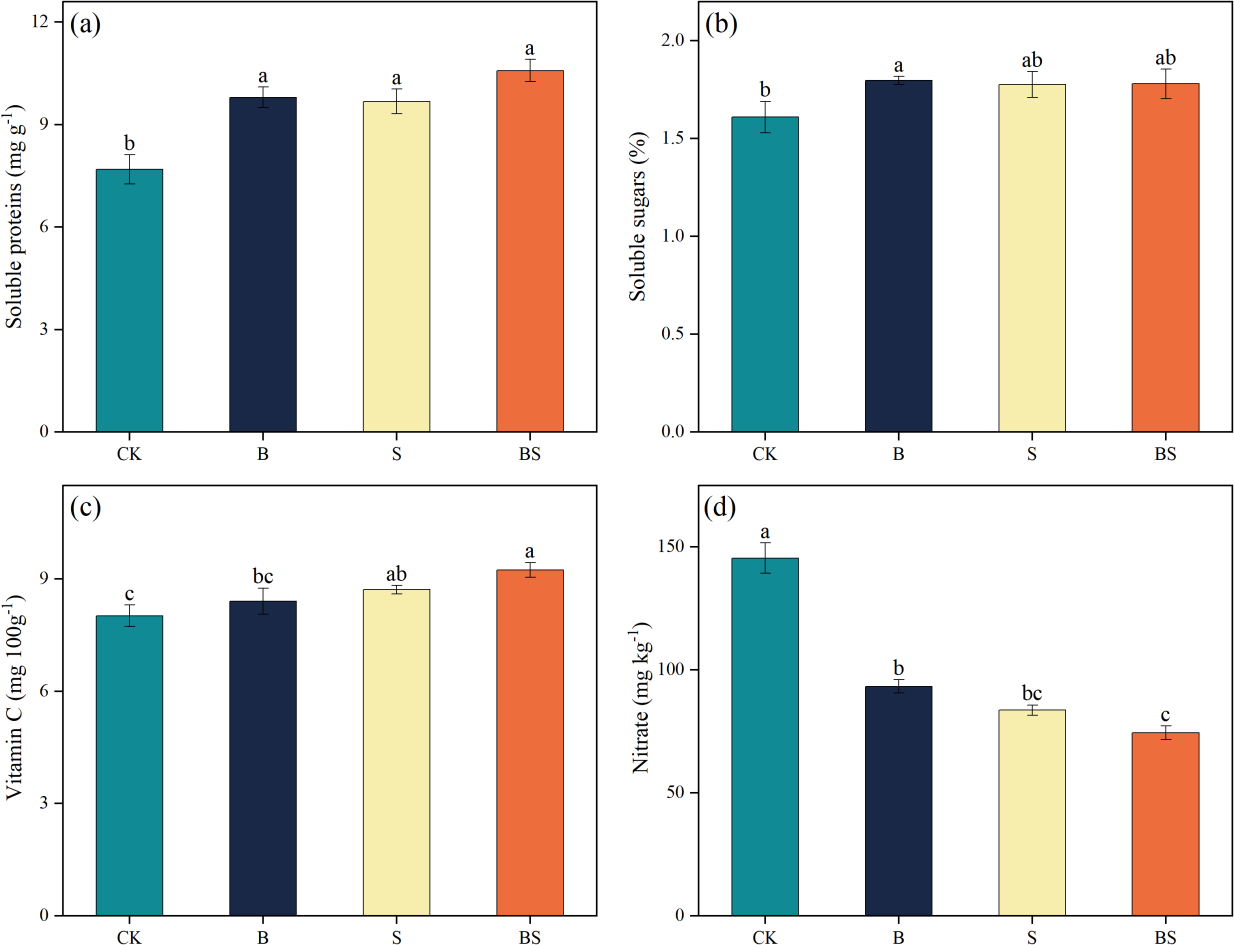
Effects of biochar and intercropping on the soluble protein **(a)**, soluble sugar **(b)**, vitamin C **(c)**, and nitrate content **(d)** in fruits of continuously cropped cucumbers. Each value represents three biological replicates (± SD) (n = 3). Different lowercase letters indicate significant differences among treatments at the *p* < 0.05 level.

Continuous cropping negatively affected cucumber fruit quality, an effect that was significantly ameliorated by biochar addition, intercropping, and particularly their combination (**Figure 2**). Compared to CK, the treatments B, S, and BS significantly increased the average content of soluble protein in cucumber fruits by 27.49%, 25.89%, and 37.73%, respectively (*p* < 0.05) (**Figure 2a**). No significant differences were observed among the three amendment treatments. Water-soluble sugar content exhibited a similar trend, with values ranking as S < B < BS (**Figure 2b**). The S and B treatments increased sugar content by 10.56% and 10.35%, respectively, compared to CK, but these increases were not statistically significant. In contrast, the BS treatment produced a significant 11.59% increase (**Figure 2b**). Vitamin C content was significantly increased by 15.25% in the BS treatment compared to CK (**Figure 2c**). All amendment treatments significantly reduced the fruit nitrate content relative to CK (145.45 mg kg^-^¹), with values decreasing to 83.65 (B), 93.32 (S), and 74.44 mg kg^-^¹ (p < 0.05; **Figure 2d**).

### 3.3 Effects of biochar and intercropping on soil phenolic

**Figure 3:**
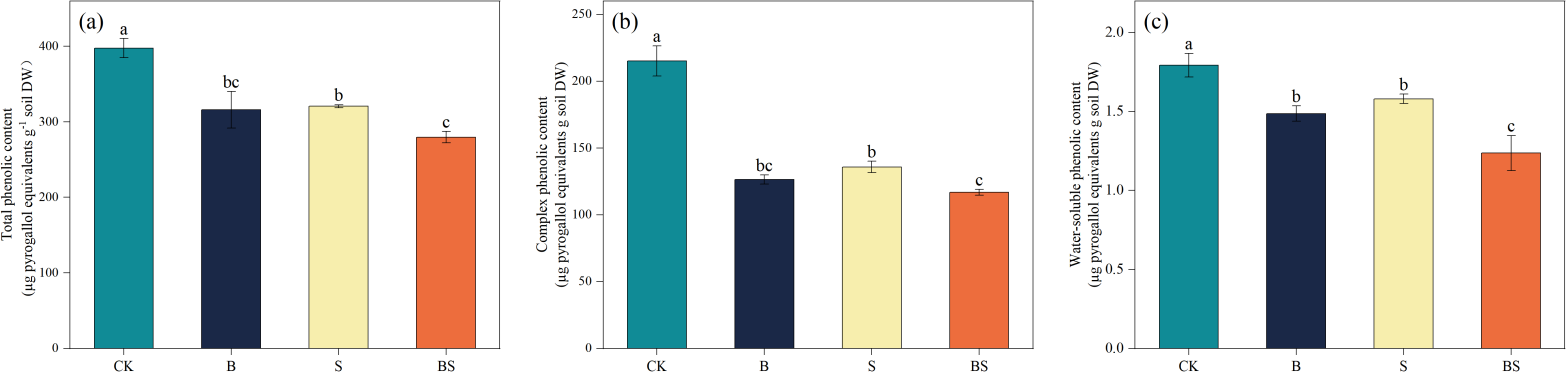
Effects of biochar and intercropping on total phenolics **(a)**, complex phenolics **(b)**, and water-soluble phenolics **(c)** in continuously cropped soils. Each value represents three biological replicates (± SD) (n = 3). Different lowercase letters indicate significant differences among treatments at the *p* < 0.05 level.

The application of biochar and intercropping significantly reduced the content of phenolic compounds in the soil (**Figure 3**). Total phenolic acid content in the cucumber rhizosphere soil was significantly decreased in all treatments relative to CK (p < 0.05; **Figure 3a**). The greatest reduction (29.66%) was observed in the BS treatment, although this value was not statistically different from that in the S treatment. The content of complex phenolics in cucumber roots was significantly reduced by 41.27% (B), 36.89% (S), and 45.73% compared to CK (**Figure 3b**). Similarly, the water-soluble phenolic content was significantly reduced by 17.10% (B), 11.90% (S), and 31.04% (**Figure 3c**).

### 3.4 Effects of biochar and intercropping on soil salinity

All amended treatments significantly reduced the electrical conductivity (EC) of the cucumber rhizosphere soil compared to CK. The BS treatment resulted in the greatest reduction (14.09%), followed by B (13.41%) and S (8.86%). The concentrations of Na^+^, SO_4_²^-^, and Cl^-^ were all significantly lowered by the amendments relative to CK (p < 0.05). The reduction in SO_4_²^-^ content ranged from 25.64% to 45.45% across treatments.For Cl^-^ and Na^+^, the BS treatment showed the greatest reductions (31.75% and 27.93%, respectively), followed by the B treatment (21.76% and 21.61%). The S treatment resulted in the smallest decreases (11.24% for Cl^-^ and 16.23% for Na^+^). The reductions achieved by the B and S treatments were not significantly different from each other. In contrast, the effects on cation concentrations (Ca²^+^, K^+^, Mg²^+^) were more variable. The S treatment significantly decreased Ca²^+^ and K^+^ levels relative to CK, but had no significant effect on Mg²^+^. Conversely, the B treatment significantly increased the concentrations of all three cations. The BS combination treatment resulted in cation concentrations that were not significantly different from those in the CK soil.

### 3.5 Effects of biochar and intercropping on soil chemical properties

The application of biochar and intercropping significantly altered several soil chemical properties, including pH, SOC, TN, AP, NH_4_^+^ and NO_3_^-^ (**Table 1**). The rhizosphere soil pH increased significantly in the B (to 7.07) and BS (to 6.85) treatments compared to the control (CK, 5.57), with increments of 1.50 and 1.28 units, respectively. In contrast, the increase observed in the S treatment (to 5.93, an increase of 0.36 units) was not statistically significant. The SOC content was significantly increased by the B and BS treatments compared to CK. In contrast, the S treatment had no significant effect on SOC. Interestingly, TN content was significantly increased by all treatments. Conversely, NO_3_^-^ content was significantly reduced compared to CK (p < 0.05). Meanwhile, no significant differences were observed in the TP content among all treatments. AP increased significantly by 32.08%, 13.85%, and 34.03% respectively compared to CK. Similarly, the AK content was significantly higher than CK only in the B and BS treatments (p < 0.05).

**Table 1:** Effects of biochar and intercropping on the chemical properties of continuously cropped soils.

Treatments	pH(water)	SOC (g kg^-1^)	TN (g kg^-1^)	TP (g kg^-1^)	AP (mg kg^-1^)	AK(mg kg^-1^)	NH_4_^+^(mg kg^-1^)	NO_3_^-^ (mg kg^-1^)
CK	5.57 ± 0.11c	8.65 ± 0.57c	1.63 ± 0.02b	1.46 ± 0.06a	85.94 ± 4.07c	246.05 ± 4.14b	16.68 ± 1.15a	93.60 ± 3.33a
B	7.07 ± 0.20a	11.43 ± 1.02b	1.79 ± 0.03a	1.47 ± 0.05a	113.52 ± 4.17a	280.15 ± 13.66a	13.68 ± 0.45ab	44.71 ± 2.67c
S	5.93 ± 0.12b	9.47 ± 0.57bc	1.79 ± 0.05a	1.43 ± 0.11a	97.85 ± 1.75b	272.08 ± 3.57ab	15.23 ± 0.37ab	60.45 ± 2.18b
BS	7.28 ± 0.05a	14.52 ± 0.85a	1.85 ± 0.04a	1.48 ± 0.03a	115.19 ± 4.75a	288.30 ± 14.15a	12.92 ± 0.35b	40.95 ± 2.27c

CK, Control; B, Biochar amendment; S, Intercropping with *Paspalum vaginatum*; BS, Biochar amendment combined with intercropping *Paspalum vaginatum*. Each value represents three biological replicates (± SD) (n = 3). Different lowercase letters indicate significant differences among treatments at the *p* < 0.05 level.

### 3.6 Effects of biochar and intercropping on soil bacterial diversity and community structure

**Figure 4:**
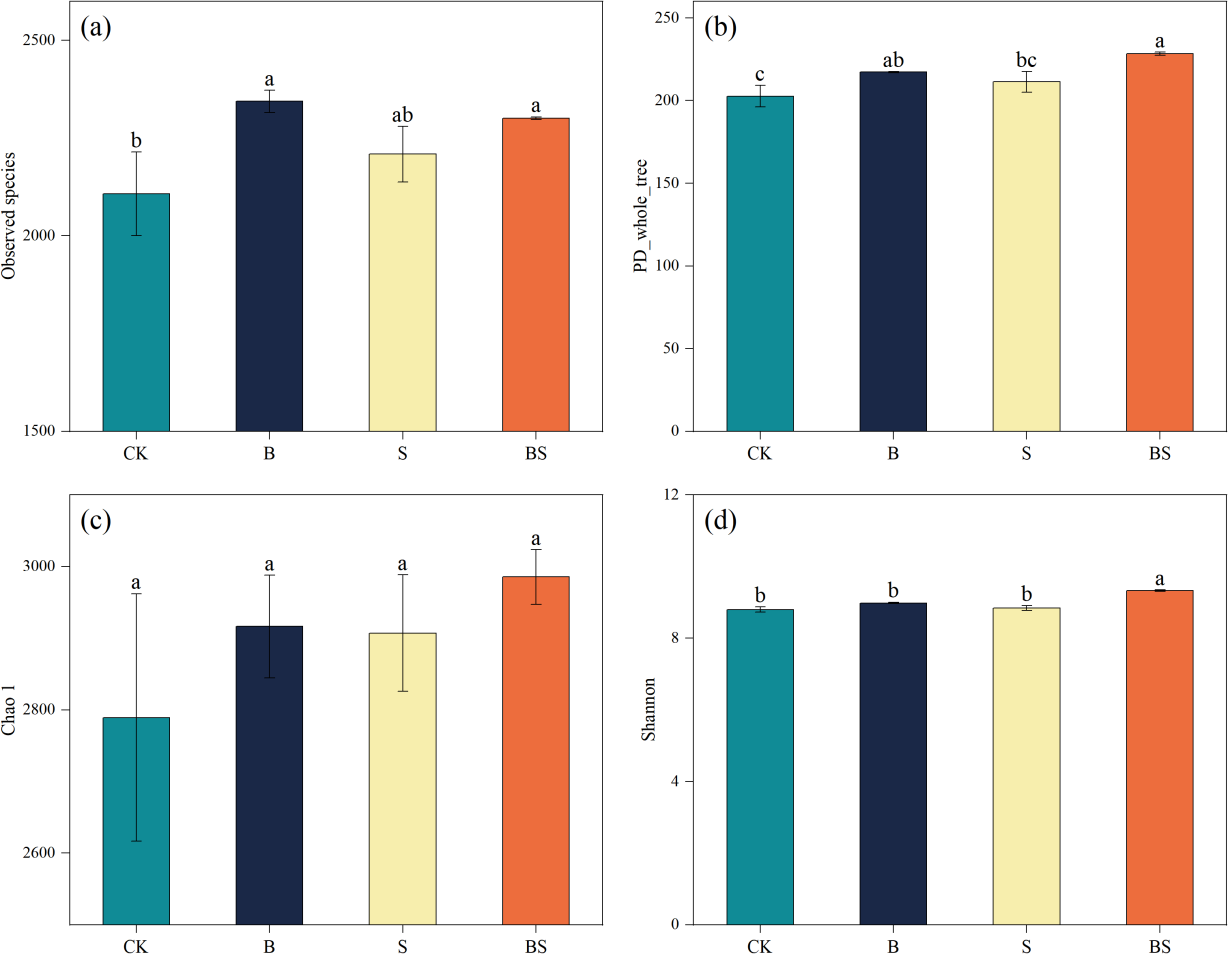
Effects of biochar and intercropping on soil bacterial observed species **(a)**, PD whole tree **(b)**, Chao1 **(c)**, and Shannon index **(d)**. Each value represents three biological replicates (± SD) (n = 3). Different lowercase letters indicate significant differences among treatments at the *p* < 0.05 level.

**Figure 5:**
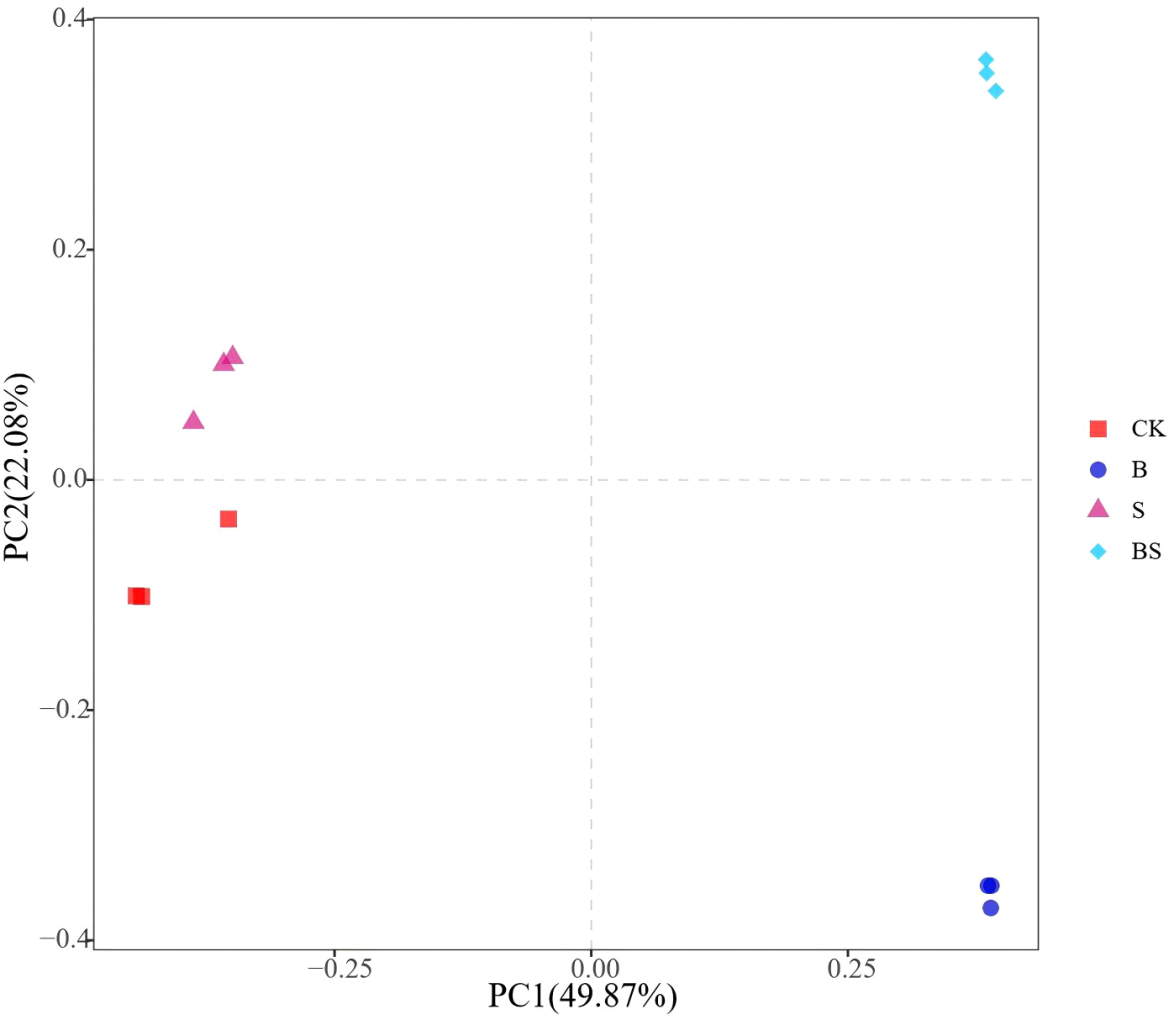
Structure of soil bacterial communities affected by biochar and intercropping. Bacterial community analysis based on Bray-Curtis dissimilarity. Different colored shapes represent different treatments. The distance between samples represents the degree of difference.

The CK treatment resulted in the lowest observed species richness, which was significantly lower than that in the B and BS treatments (p < 0.05; **Figure 4a**). A similar trend was observed for phylogenetic diversity (PD), with CK also being significantly lower than B and BS (**Figure 4b**). In contrast, the Chao1 index did not differ significantly among treatments (**Figure 4c**). The Shannon index, a measure of community diversity, was highest in the BS treatment and was significantly greater than in all other treatments (**Figure 4d**). Principal coordinates analysis (PCoA) based on OTU profiles (97% similarity) revealed clear separation in bacterial community structure among the treatments. The PCoA plot showed a clear separation along PC1, with the B and BS treatments clustering separately from the CK and S treatments (**Figure 5**).

### 3.7 Effects of biochar and intercropping on the composition of soil bacterial communities

**Figure 6:**
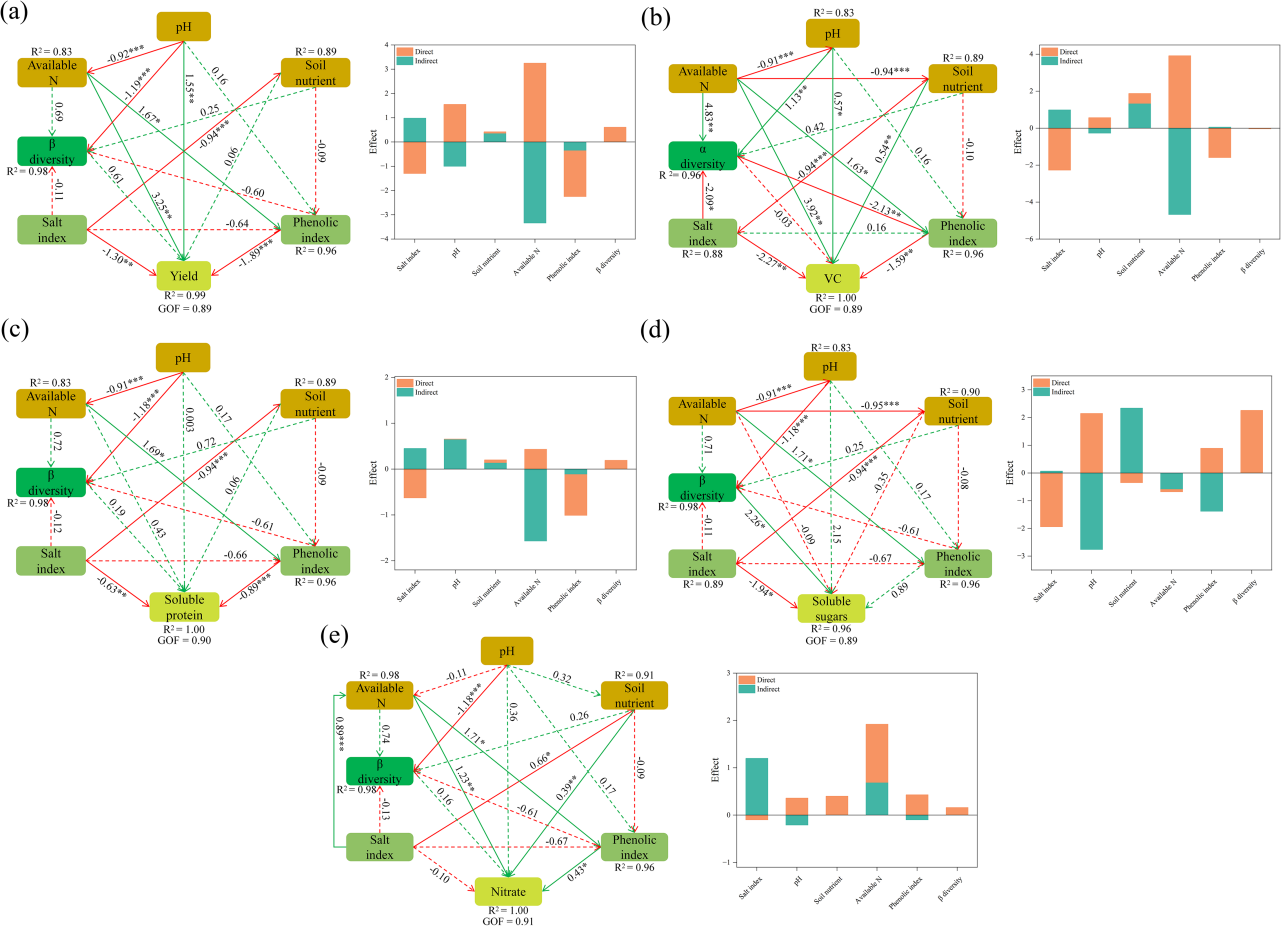
The partial least-squares path model (PLS-PM) showing the effects of biochar and intercropping on cucumber yield **(a)**, soluble sugar **(b)**, soluble protein **(c)**, vitamin C (Vc) **(d)**, and nitrate **(e)**. Green and red solid lines represent significant positive and negative effects, respectively, while dashed lines indicate non-significant effects.

The composition of soil bacterial communities was significantly altered by the amendments at the phylum level (**Figure 6**). Despite treatment effects, the overall phylum-level profile was similar across all samples. The dominant phyla, collectively accounting for 92.87% of sequences, were *Proteobacteria*, *Bacteroidota*, *Patescibacteria*, *Gemmatimonadota*, *Chloroflexi*, *Firmicutes*, *Acidobacteriota*, *Actinobacteriota*, and *Cyanobacteria*. The relative abundance of *Proteobacteria* was significantly higher in the CK treatment than in the BS treatment. In contrast, the relative abundances of *Gemmatimonadota*, *Bacteroidota*, and *Chloroflexi* were lower in the CK treatment than in the amended treatments. The S treatment was associated with the highest relative abundances of *Patescibacteria* and *Cyanobacteria*. The relative abundance of *Actinobacteriota* was significantly lower in all amended treatments (B, S, BS) compared to the CK treatment.

### 3.8 Relationships between soil properties and cucumber yield and quality

The partial least-squares path model (PLS-PM) was used to explore the dominant factors affecting cucumber yield and quality (**Figure 6**). Cucumber yield was significantly affected by soil variables, with soil pH having a substantial positive impact, while available N, salt properties, and phenolic acids had significant negative effects (**Figure 6a**). In contrast, the influence of soil bacterial diversity on cucumber yield was relatively minor. Unlike yield, soil bacterial diversity and salt properties had direct and significant positive and negative effects on soluble sugar content, respectively (**Figure 6b**). Soil pH and nutrient availability indirectly enhanced the soluble sugar content of cucumbers, whereas available N had the opposite effect. Similar to soluble sugar, available N, salt properties, and phenolic acids were negatively correlated with soluble protein and vitamin C content in cucumbers, while pH had the opposite effect. Additionally, Vc content was significantly influenced by positive effects from microbial diversity and soil nutrient availability, and a negative effect from salt properties (**Figures 6c, d**). However, microbial diversity was not a primary driver of nitrate content in cucumbers. Instead, nitrate content in cucumbers was significantly and positively influenced by available N and phenolic acids (**Figure 6e**).

## 4 Discussion

To address the progressive degradation of greenhouse soil health caused by continuous cropping and to promote sustainable agricultural production, various techniques such as soil fumigation, soil replacement, and grafting have been proposed to mitigate the obstacles associated with continuous cropping (Ding et al., 2021). However, these methods may increase production costs, cause environmental damage, and reduce crop quality, whereas the use of biochar or intercropping avoids these disadvantages (Jesus et al., 2024; Wang et al., 2021a). Therefore, to reduce improvement costs and minimize environmental impact, we integrated biochar with intercropping to investigate the mechanisms through which this combination alleviates soil constraints in continuous cropping systems. In this study, the combined application of biochar and intercropping significantly alleviated the degradation of soil quality caused by continuous cropping obstacles. The application of biochar, both alone and in combination with intercropping, modified the soil’s biochemical properties, thereby directly or indirectly enhancing the yield and quality of cucumbers in continuously cropped soil.

### 4.1 Effect of biochar and intercropping on soil physicochemical properties

Long-term continuous cropping, coupled with excessive and imbalanced fertilizer inputs, leads to decreased soil pH, secondary salinization, and nutrient imbalances (Gong et al., 2024; Zheng et al., 2021). These phenomena are recognized as major factors contributing to continuous cropping obstacles in greenhouse production systems (Bai et al., 2020; Zhang et al., 2021). Consequently, persistent soil degradation markedly diminishes the productive capacity of agricultural land and threatens the sustainability of crop production (Li et al., 2024; Yan et al., 2024). In this study, owing to its inherent alkalinity and high organic carbon content, biochar application—both alone and combined with intercropping—effectively increased soil pH and SOC content (**Table 1**) (Liu et al., 2014; Zhang et al., 2010). Previous research has demonstrated a negative correlation between soil available N content and pH (Liu et al., 2023b). This relationship may be attributed to enhanced ammonification, which consumes H^+^ ions and consequently elevates soil pH (Xu et al., 2006). These processes contribute to creating a more favorable soil environment for plant growth. In continuous cropping systems, the availability of phosphorus (AP) and potassium (AK) often declines, whereas available N frequently accumulates, resulting in severe nutrient imbalances (Li et al., 2017; Liu et al., 2023b). Our findings showed that biochar amendment and intercropping can ameliorate soil quality by enhancing AP and AK availability, a phenomenon corroborated by previous studies (**Table 1**) (Wang et al., 2024c; Zhou et al., 2021). Interestingly, both biochar application and intercropping also significantly reduced the soil NO_3_^-^ content (**Table 1**). The observed reduction in soil available N could potentially be attributed to the strong adsorption capacity of biochar, which may immobilize mineral nitrogen, coupled with the increased AN uptake by the intercropped plants, but may also alter the N-cycling mediated by microbes, such as ammonia-oxidizing bacteria (AOB) and archaea (AOA) (Lehmann et al., 2021; Xiang et al., 2024). Further research into microbe-involved nitrogen cycling processes is needed to reveal the mechanisms of N cycling mediated by biochar. The reduction in available N, particularly in the form of NO_3_^-^, might initially appear detrimental, excessively high levels of NO_3_^-^ are in fact phytotoxic—a common scenario in greenhouse production systems (Zhang et al., 2021). The strong effect of biochar or intercropping in reducing available N content may be more effective when applied to greenhouse continuous cropping soils degraded by excessive nitrogen fertilizer application. Thus, both biochar and intercropping play crucial roles in rebalancing soil nutrient dynamics (Hauggaard-Nielsen and Jensen, 2005). Furthermore, compared to CK, the soil phenolic acid content decreased significantly across all amendment treatments, with the most substantial reduction observed in the BS treatment (**Figure 3**). This indicates that both biochar and intercropping directly or indirectly influence the soil micro-ecological environment, ultimately affecting cucumber yield and quality. The mitigation of autotoxic effects on cucumbers by biochar may be attributed to its high porosity, substantial adsorption capacity, and large specific surface area, which likely facilitate the adsorption of phenolic acids. Additionally, biochar-induced alterations in the soil microbial community might contribute to the degradation of these autotoxic substances (Yang et al., 2019a). Similarly, intercropping reduces phenolic acid levels,which may be due to the root exudates from *Paspalum vaginatum* that enhance nutrient availability and stimulate microbial activity, thus alleviating their autotoxic impact on cucumbers (Li et al., 2020). In conclusion, our results indicate that integrating biochar with intercropping may serve as a promising strategy for improving soil physicochemical properties and mitigating continuous cropping obstacles in intensive cultivation systems, warranting further validation under field conditions.

### 4.2 Effect of biochar and intercropping on soil salinity

Soil properties were influenced by the application of biochar and intercropping. As a halophyte, *Paspalum vaginatum* exhibits a high degree of salt tolerance, with certain ecotypes capable of withstanding salt concentrations equivalent to 80% of that in seawater (Lee et al., 2004). Consistent with its halophytic nature, intercropping with *Paspalum vaginatum* significantly reduced the levels of soluble salt ions and soil electrical conductivity (EC) (**Table 2**). This reduction is likely attributable to the pronounced capacity of *Paspalum vaginatum* to uptake and sequester salt ions, accumulating them in both root and shoot tissues (Guo et al., 2016; Hu et al., 2020). This mechanism is supported by similar findings for other species; for instance, the high salt-absorption potential of alfalfa has been shown to reduce soil salt accumulation and consequently lower EC (Su et al., 2024). Similarly, biochar ameliorates salinity through distinct mechanisms. Its surface, characterized by both positive and negative charges and a diversity of functional groups, facilitates the adsorption of various salt ions, thereby reducing soil salinity (Farhangi-Abriz and Ghassemi-Golezani, 2021; Rajapaksha et al., 2016). Our results showed that biochar application significantly reduced the concentrations of Na^+^, SO_4_²^-^, and Cl^-^, while concurrently increasing the levels of Ca²^+^ and Mg²^+^ (**Table 1**) (Jin et al., 2024; Zhang et al., 2022a). These observed ionic shifts are consistent with a potential cation exchange process, wherein the release of divalent cations (Ca^2+^ and Mg^2+^) from biochar could promote the displacement of Na^+^ from soil exchange sites into the soil solution, perhaps facilitating its leaching from the root zone (Agbna et al., 2017; Cui et al., 2021). Furthermore, the combined application of biochar and intercropping elicited a more pronounced reduction in key soil salinity indicators compared to biochar alone, pointing to a potential synergistic effect. This enhanced efficacy could be due to several interconnected factors. For instance, biochar-induced improvements in soil physical properties, such as porosity and moisture retention (Qian et al., 2020; Wang et al., 2022a), likely create a more favorable rhizosphere environment. This, in turn, may bolster the capacity of *Paspalum vaginatum* to absorb, sequester, or tolerate salts. Additionally, modifications to the microbial community by biochar or changes in root exudation patterns due to intercropping might also contribute to this synergistic salinity mitigation.

**Table 2:** Effects of biochar and intercropping on propertiess of continuously cropped soils.

Treatments	EC (mS cm^-1^)	Na^+^(mg kg^-1^)	Ca^2+^(mg kg^-1^)	K^+^ (mg kg^-1^)	Mg^2+^ (mg kg^-1^)	SO_4_^2-^ (mg kg^-1^)	Cl^-^ (mg kg^-1^)
CK	1.47 ± 0.02a	189.53 ± 9.38a	150.33 ± 3.30b	89.21 ± 2.92b	51.57 ± 3.66b	495.58 ± 46.42a	159.02 ± 3.43a
B	1.27 ± 0.22c	148.76 ± 4.03bc	193.98 ± 4.84a	103.56 ± 4.20a	65.63 ± 3.07a	270.34 ± 4.12c	124.42 ± 9.52c
S	1.34 ± 0.02b	158.76 ± 10.34b	113.53 ± 7.73c	72.93 ± 5.46c	49.00 ± 4.33b	368.50 ± 25.92b	141.15 ± 7.61b
BS	1.26 ± 0.02c	136.59 ± 5.15c	167.53 ± 12.48b	85.84 ± 3.79b	54.77 ± 5.24ab	306.02 ± 14.19bc	108.53 ± 5.13bc

CK, Control; B, Biochar amendment; S, Intercropping with *Paspalum vaginatum*; BS, Biochar amendment combined with intercropping *Paspalum vaginatum*. Each value represents three biological replicates (± SD) (n = 3). Different lowercase letters indicate significant differences among treatments at the *p* < 0.05 level.

### 4.3 Effect of biochar and intercropping on the yield and quality of cucumbers

Continuous cropping obstacles inhibit crop growth and severely reduce crop yield and quality (Li and Cai, 2016; Liao et al., 2018). Previous studies have explored the potential of biochar application or intercropping to alleviate continuous cropping obstacles and thereby improve crop yield and quality (Hu et al., 2020; Zhang et al., 2023). However, it remains unclear whether the combined application of biochar and intercropping exerts a synergistic effect on improving crop growth under continuous cropping systems and on enhancing soil health. Enhancing crop yield and quality is a major objective in agricultural production (Souza et al., 2025; Wu et al., 2025). In this study, the combined application of biochar and intercropping significantly enhanced cucumber growth and increased yield (**Figure 1**). Studies have indicated that biochar can alleviate soil degradation induced by continuous cropping and excessive fertilization, while also enhancing crop yields (Cui et al., 2021; Zhang et al., 2023). Biochar is rich in mineral elements and possesses a large specific surface area and high adsorption capacity. These properties can reduce NO_3_^-^ but increase AP and AK, thereby helping to balance soil nutrient status (Li et al., 2023). Moreover, biochar application can improve the soil microbial environment and enhance microbial activity, thereby promoting plant growth (Li et al., 2023; Lv et al., 2023). The positive effects of intercropping on crop yield can be attributed to multiple mechanisms. Studies have shown that intercropping can enhance the availability of soil nutrients, promote leaf photosynthesis, and facilitate fruit set and development (Wang et al., 2022b). Furthermore, intercropping with halophytes can ameliorate soil salinity conditions, thereby providing a more favorable low-salt environment for crop growth (Simpson et al., 2018). Additionally, our results showed that the combined application of biochar and intercropping increased the contents of soluble sugars, soluble proteins, and vitamin c in cucumber fruits, while significantly reducing fruit nitrate content (**Figure 2**). This indicates that both the organic amendment (biochar) and intercropping have positive effects on fruit quality. Due to its high stability, biochar allows for the slow and continuous release of nutrients, which promotes balanced nutrient uptake and utilization by crops, ultimately enhancing fruit quality (Naeem et al., 2018; Zhao et al., 2017). Previous studies have reported similar findings: in cucumber continuous cropping systems, biochar amendment improves the crop growth environment by mediating soil nutrient balance and reducing salinity, thereby promoting growth and increasing both yield and quality (Wang et al., 2021a; Zhou et al., 2021). Consistent with our findings, studies have shown that intercropping with halophytes also improves soil physicochemical properties and reduces soil salinity, thereby creating a more favorable growth environment for crops (Jurado et al., 2024; Slatni et al., 2024).

### 4.4 Effect of biochar and intercropping on soil bacterial communities

The application of biochar and intercropping significantly influenced soil microbial diversity and composition (**Figures 4**, **5**), which are crucial for nutrient cycling and plant health in agricultural ecosystems. Soil acidification and salinization resulting from continuous cropping profoundly alter microbial community structure and suppress their metabolic activities (Ji et al., 2022; Marcheva et al., 2024). The community of microorganisms is primarily impacted by nutrients, pH, and salinity (Banda et al., 2021). In this study, the combined application of biochar and intercropping not only significantly increased soil microbial diversity (**Figures 4**, **5**), but also induced a profound functional reshaping of the microbial community.

The LEfSe analysis provided tangible evidence for this structural shift, identifying specific bacterial taxa that were significantly enriched in the BS treatment (**Supplementary Figure S1**). These biomarkers included families within the Actinobacteriota, such as Micromonosporaceae and Nocardiopsaceae, which are renowned for their capacity as prolific producers of antibiotics and hydrolytic enzymes (Mitra et al., 2021). Crucially, this taxonomic shift was directly reflected in the functional potential predicted by PICRUSt2 (**Supplementary Figure S2**). The BS treatment exhibited a significant enhancement in the “Neomycin, kanamycin and gentamicin biosynthesis” pathway. This coherent finding strongly suggests that the BS combination does not merely increase microbial abundance but specifically enriches for keystone taxa within the actinobacterial community and upregulates their antibiotic synthesis potential, thereby constructing a more robust biological defense line against soil-borne pathogens—a core challenge in continuous cropping systems.

**Figure 7:**
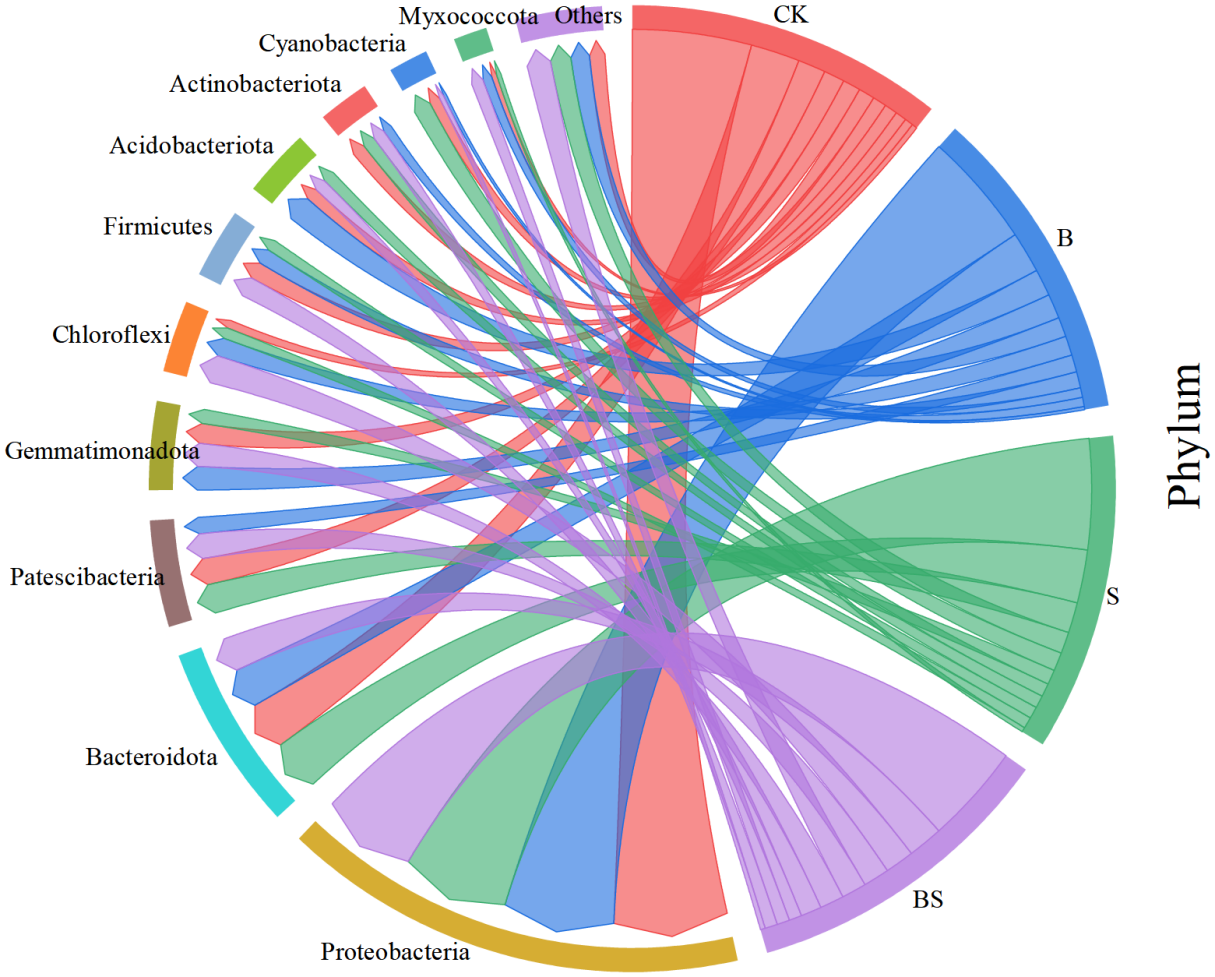
Relative abundance of bacterial phyla in soils treated with biochar and intercropping.

Concurrently, the PICRUSt2 results revealed a significant reduction in “Bacterial chemotaxis” under BS treatment (**Supplementary Figure S2**). This functional shift holds important ecological implications, as chemotaxis is an energy-costly behavior for microorganisms seeking resources in oligotrophic or stressful environments (Wadhams and Armitage, 2004). Its suppression likely indicates that the BS-amended soil offers a more favorable and less stressful microenvironment with improved nutrient accessibility (**Tables 2**, **1**), allowing microbes to allocate more energy from motility to growth and beneficial metabolite production. This notion is further supported by the significant enrichment of “D-Alanine metabolism” and “D-Glutamine and D-glutamate metabolism” pathways, which are integral to bacterial cell wall synthesis and osmotic stress regulation (Cava et al., 2011; Waldemar et al., 2008), indicating a microbial community with enhanced growth activity and resilience under the BS regime. This finding aligns with previous studies showing that while salinity adversely affects bacterial community composition, the application of biochar or intercropping under salt stress mitigates these effects and exerts favorable effects on bacterial structure (Szoboszlay et al., 2019; Xie et al., 2022). These practices likely enhance microbial diversity by supplying nutrients and creating additional ecological niches for beneficial microorganisms (Fu et al., 2017). Furthermore, the increased diversity may enhance functional resilience, allowing the microbial community to maintain activity under a wider range of environmental conditions through niche adaptation and functional redundancy (Garcia-Garcia et al., 2019). Therefore, by enhancing microbial diversity, the biochar-intercropping combination fosters a more robust soil microenvironment and ensures the stability of ecosystem functions mediated by microbes. PCoA shows that the microbial communities of CK and BS treatments are clearly separated (**Figure 5**). This shift in community composition is consistent with previous findings that plant-soil interactions can greatly alter microbial community structures (Jin et al., 2020; Zhou et al., 2021). These community changes are functionally significant, as rhizosphere microbes are key agents in plant nutrient acquisition, soil structure formation, and the production of regulatory exometabolites (Chi et al., 2023; Tong et al., 2024). Thus, by reshaping the soil microbial composition, biochar and intercropping indirectly promote plant growth and health (Wang et al., 2024a). Specifically, the BS treatment significantly increased the relative abundance of several bacterial phyla, including *Actinobacteriota*, *Gemmatimonadota*, and *Chloroflexi* (**Figure 7**). The proliferation of these oligotrophic taxa may be driven by the moderated soil nitrogen availability (Wang et al., 2024b). These phyla are integral to soil nutrient cycling, facilitating the transformation of carbon, nitrogen, and phosphorus through the secretion of various enzymes and metabolites, thereby promoting plant growth (Morrissey et al., 2023; Mujakic et al., 2023; Rao et al., 2022). The overall improvement in soil nutrient supply capacity (Bandara et al., 2022) and the elevated pH are likely contributing factors to the success of these bacterial groups. It is noteworthy that *Acidobacteriota*, which are often considered acidophilic, dominated the CK treatment with lower pH (**Table 1**). Previous studies suggested that *Acidobacteria* played an important role in biogeochemical cycling of carbon and consequently might be adaptable to the environment of large variety of carbon sources present in biochar (Zhong et al., 2024). However, the soil showed a more alkaline environment after biochar amendment, which is not favorable for *Acidobacteria*, a phylum of bacteria usually being acidophilic (Lehmann et al., 2011). As a result, the abundance of *Acidobacteria* decreased with biochar addition. Notably, *Actinobacteria* not only produce antibiotics to suppress plant pathogens and decompose organic matter but also reduce bacterial wilt in plants. They exhibit accelerated growth in nutrient-rich environments (Lee et al., 2021), and the application of biochar improves soil conditions, thereby stimulating the proliferation of these beneficial microorganisms. Biochar itself provides a physical refuge and a slow-release carbon source for microorganisms, while the root exudates from the intercropped *Paspalum vaginatum* supply easily degradable carbon sources for specific microbial taxa. It is plausible that the release of phenolic compounds from the aromatic carbon structures of biochar, facilitated by *Actinobacteria*, may further stimulate the abundance and activity of microbial groups capable of decomposing phenolic acids (Kolton et al., 2017), thereby potentially alleviating their adverse effects on plants. Furthermore, the improved treatments reduced the relative abundance of potentially pathogenic *Proteobacteria* (Tan et al., 2017), while enriching for beneficial genera such as *Gemmatimonas*, which is known to suppress *Fusarium* wilt and promote plant growth (Shen et al., 2024; Zheng et al., 2024; Zhong et al., 2024). Furthermore, studies have shown that biochar and intercropping can alleviate the inhibitory effects of salt ions on microorganisms, promoting the growth and metabolism of dominant salt-tolerant bacteria such as *Bacteroidota* and *Actinobacteriota* (Wang et al., 2024d). This shift in the microbial balance, from a community dominated by potential pathogens and acidophiles (e.g., *Acidobacteriota* in low-pH CK) to one enriched with beneficial taxa identified by LEfSe (e.g., antibiotic-producing *Actinobacteriota*) and functionally equipped for nutrient cycling and stress resistance (as per PICRUSt2), likely contributed significantly to the observed enhancement in plant growth by simultaneously mitigating soil-borne diseases and improving soil fertility.

Our PLS-PM analysis identified the amelioration of soil physicochemical properties, not bacterial diversity, as the primary driver for enhanced cucumber yield. This can be attributed to several factors: First, the rapid alleviation of abiotic stressors (e.g., Al^3+^ toxicity, salinity) via increased pH and adsorption provided immediate physiological benefits, outweighing the more gradual effects of microbial shifts (Lehmann et al., 2011). Second, biochar and intercropping primarily improved the physicochemical environment, which subsequently steered the microbial community as a secondary effect (Lehmann et al., 2011). Third, under the severe abiotic stress of the degraded soil, directly mitigating these constraints was a prerequisite for plant growth, a role in which physicochemical amelioration is inherently faster and more direct than microbial mediation (Ranjbari et al., 2022). Consequently, the direct improvement of soil physicochemical properties emerged as the dominant mechanism in this short-term pot study.

To identify the key drivers influencing cucumber yield and quality, a partial least-squares path modeling (PLS-PM) analysis was performed (**Figure 6**). The results indicated that soil pH and soil nutrition play significant positive roles in enhancing cucumber yield and quality, while they are negatively correlated with soil properties, available N content, and phenolic acid concentrations. The PLS-PM results suggest that biochar amendment and intercropping likely enhance cucumber yield and quality primarily by mitigating these soil stressors (e.g., salinity, excessive AN, and phenolic acids). This finding is consistent with previous studies reporting that soil amendments and intercropping systems can alleviate soil salinity and phenolic acid toxicity, thereby promoting crop growth (Hu et al., 2020; Zhou et al., 2021). In contrast, microorganisms have a relatively minor impact on cucumber yield and fruit quality. Therefore, we propose that the enhancement of cucumber yield and quality is primarily a result of the direct improvement of soil physicochemical properties by biochar and intercropping, rather than being predominantly mediated through changes in the microbial community.

While this pot experiment provides compelling evidence for the synergistic benefits of combining biochar with halophyte intercropping under controlled conditions, it is important to acknowledge its limitations. The pot scale restricts the extrapolation of our findings directly to field-scale agricultural systems. Field environments are subject to greater heterogeneity in soil properties, climate variability, and larger-scale management practices, which could modulate the efficacy of the combined treatment. Therefore, our results should be interpreted as a proof-of-concept, demonstrating the strong potential of this integrated strategy for mitigating continuous cropping obstacles. The promising outcomes observed here justify and necessitate future long-term field trials to validate these findings, optimize application rates (e.g., biochar dosage, intercropping density), and assess the economic viability and large-scale practicality of this approach for sustainable greenhouse vegetable production.

## 5 Conclusions

The application of biochar, both alone and in combination with intercropping, effectively improved soil health, leading to enhanced yield and quality of cucumbers. Both biochar and intercropping significantly increased soil pH, SOC, TN, AP, and AK. In contrast, these treatments significantly reduced the soil NO_3_^-^ content. Additionally, the concentrations of phenolic acids and soil salinity were significantly reduced. Among all treatments, the combined application of biochar and intercropping proved to be the most effective strategy for alleviating soil acidification and salinity, reducing phenolic acids, regulating nutrient balance, increasing SOC, enhancing bacterial diversity, and ultimately improving cucumber yield and quality. Partial least-squares path modeling (PLS-PM) results demonstrated that soil pH and nutrient availability had direct positive effects on cucumber yield and quality, whereas phenolic acids, salinity, and excessive available nitrogen exerted significant negative effects. In conclusion, our pot study provides evidence that the integrated application of biochar and intercropping holds promise as a sustainable strategy for mitigating continuous cropping obstacles in cucumber production. These findings, obtained under controlled conditions, offer a proof-of-concept demonstrating the potential of this combined approach to synergistically improve soil health and crop performance. To translate this potential into practical agricultural practice, the long-term efficacy and economic viability of this strategy must be validated through well-designed field trials.

## Data Availability

The datasets presented in this study can be found in online repositories. The names of the repository/repositories and accession number(s) can be found below: https://www.ncbi.nlm.nih.gov/, SAMN51751506.
