# Supplementary material for: Combined application of biochar and halophyte intercropping enhances cucumber yield and quality by ameliorating soil properties in a continuous cropping system
Source: Front Plant Sci. 2025 Nov 20;16:1711099. doi: 10.3389/fpls.2025.1711099 (PMC12675486; doi:10.3389/fpls.2025.1711099)
Supplement: Supplementary file 1 [file DataSheet1.pdf]

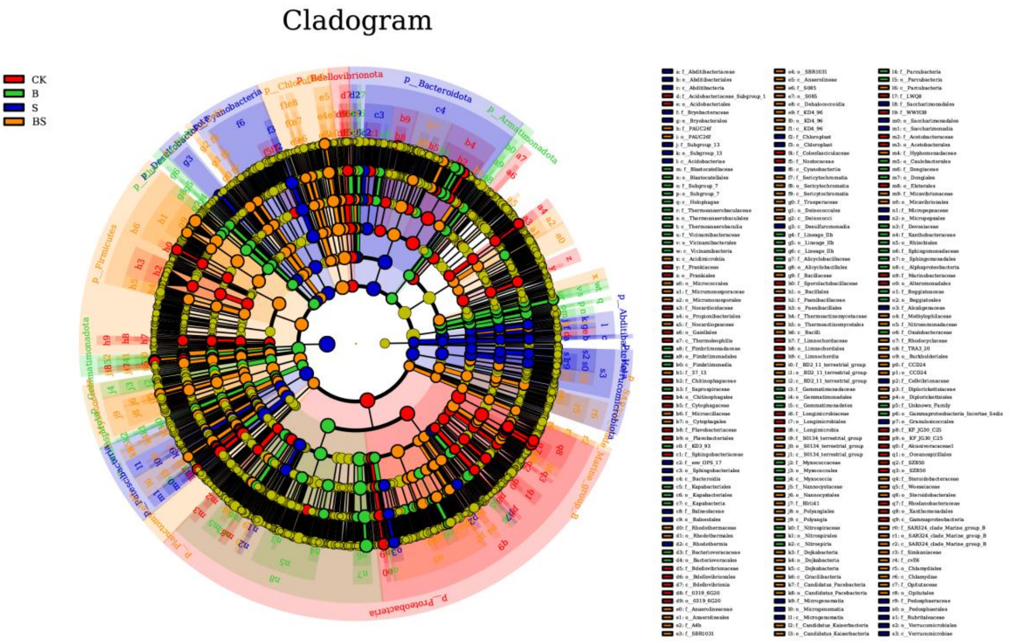

Fig. S1. Dendrograms of enriched biomarkers in different treatments analyzed by Linear Discriminant Analysis (LEfSe).

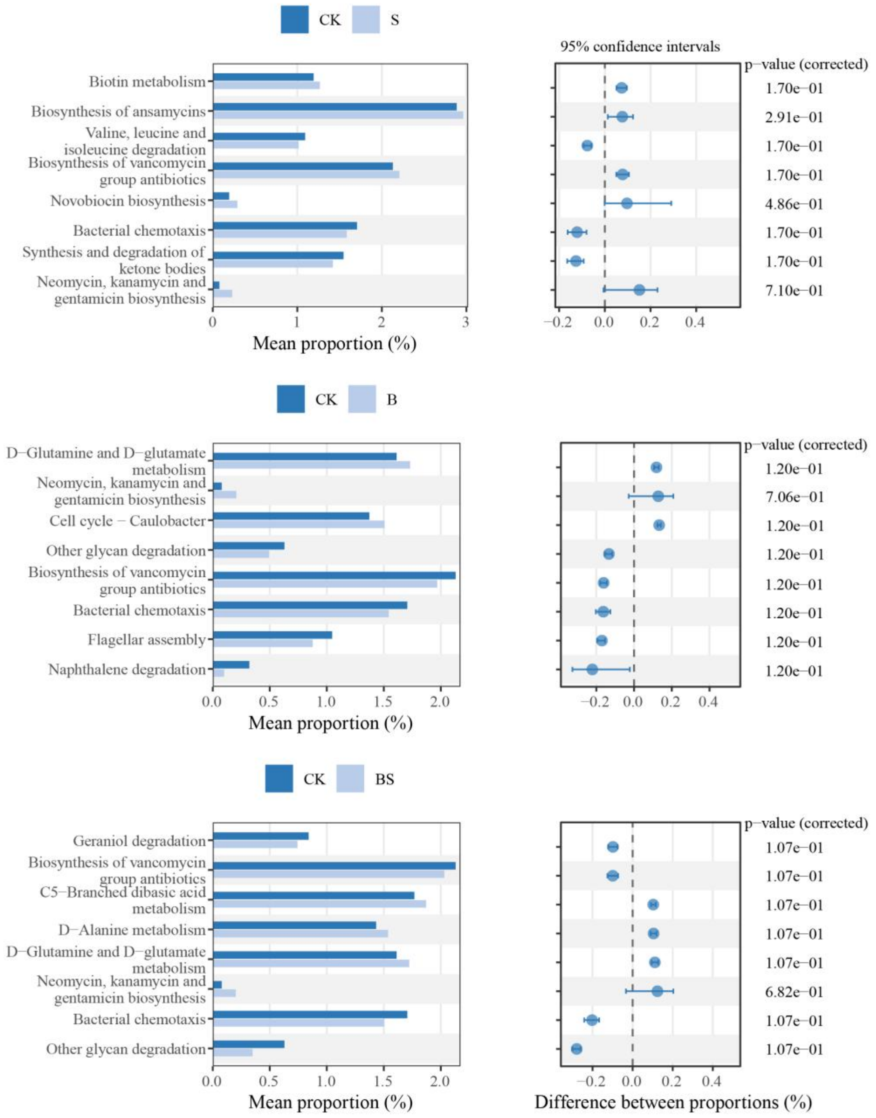

Fig. S2. Impact of Different Treatments on Functional Profiling of Soil Microbial Communities Based on the KEGG Database Using PICRUSt2.
